# AMR Sign - An Arthroscopic S-shaped Fold Signifying Adequate Medial Meniscus Repair

**DOI:** 10.5704/MOJ.2307.003

**Published:** 2023-07

**Authors:** AM Rajani, UA Shah, ARS Mittal, S Gupta, R Garg, AA Rajani, M Punamiya, R Singhal

**Affiliations:** 1 Department of Orthopaedics, Breach Candy Hospital Trust, Mumbai, India; 2 Department of Orthopaedics, Surgikids Hospital, Ahmedabad, India; 3 Department of Orthopaedics, Orthopaedic Arthroscopy Knee and Shoulder Clinic, Mumbai, India; 4 Department of Orthopaedics, Galaxy Hospital, Bhopal, India; 5 Department of Orthopaedics, Canadian Specialist Hospital, Dubai, United Arab Emirates; 6 Department of Radiology, Orthopaedic Arthroscopy Knee and Shoulder Clinic, Mumbai, India; 7 Department of Physiotherapy, Orthopaedic Arthroscopy Knee and Shoulder Clinic, Mumbai, India; 8 Department of Biostatistics, Orthopaedic Arthroscopy Knee and Shoulder Clinic, Mumbai, India

**Keywords:** AMR sign, Medial meniscus tear, arthroscopic repair, all inside, adequate repair

## Abstract

**Introduction:**

The preferred management of medial meniscus tears has notably moved from meniscectomies towards repair. With a higher volume of meniscal repairs being done all across the world with every passing day, the lack of an objective and definitive sign suggesting the adequacy of its repair is daunting. The purpose of our study was to introduce a unique and novel arthroscopic sign formed after adequate repair of the medial meniscus, the AMR (Adequacy of Medial meniscus Repair) sign. We hypothesised that it is not only the objective end point for repair, but can also form the indicator for excellent clinical, functional, and radiological outcome even in the long term.

**Materials and methods:**

This was a multicentric, prospective study initiated by the corresponding author, and the findings validated subsequently by the other authors. Overall, it included 804 patients of isolated medial meniscus tear operated with arthroscopic all-inside technique between January 2014 and December 2017. Patients were segregated into three groups based on whether an S-shaped curve in the free, inner edge of the medial meniscus sign was formed post-repair, lost after further tightening, or not formed upon subjective completion of repair. All the patients were followed-up and evaluated based of medial joint line tenderness, McMurray’s test for medial meniscus, IKDC score, WOMET score, and radiologically using an MRI at the terminal follow-up.

**Results:**

The mean terminal follow-up was 42.34±4.54 months. There was significant (p<0.01) improvement in all patients at the terminal follow-up post-surgery, irrespective of the group. The group in which AMR sign was formed and maintained showed a significantly better functional outcome on terminal follow-up as well as lower failure rates compared to the other two groups.

**Conclusion:**

AMR sign is an S-shaped fold at the inner, free edge of medial meniscus, formed after an adequate repair of isolated medial meniscus tear, as viewed on arthroscopy. It is an objective sign denoting regained integrity of the collagen architecture of the medial meniscus following repair. It is also a reliable indicator of excellent long term functional, clinical, and radiological outcome and also lower failure rates in patients after arthroscopic medial meniscus repair.

## Introduction

Orthopaedic literature has come a long way in recognising the role of the meniscus as an important intra-articular structure and not merely a remnant tissue that became vestigial in the course of evolution. In addition to streamlining the transmission of force within the knee joint, the menisci also maintain the congruency of the joint, lubrication and provide joint stability^1-3^. Owing to their close conformity with the tibial plateau and the femoral condyles in all degrees of motion, the meniscus can get injured in almost all types of knee injuries irrespective of age. Without any adequate treatment, the damaged meniscus loses its function, thereby making the affected knee susceptible to early osteoarthritis^4,5^.

It not only provides stability during the anteroposterior femorotibial rolling but also transmits hoop stresses from the femur to the tibia on axial loading. This is owed to the highly specialized arrangement of collagen fibres, with a majority being circumferentially aligned (hoop structure)^6,7^. An injury to the meniscus disrupts the hoop structure leading to abnormal biomechanics between the femur and tibia, thereby altering weight-bearing kinematics. This alteration also leads to early osteoarthritis.

Magnetic resonance imaging (MRI) has long been established as the most accurate non-invasive imaging modality for the diagnosis of simple and complex meniscal tears, however, arthroscopy still remains the gold standard^8^. On arthroscopy, identification of a meniscus tear can be challenging at times. The intact medial meniscus can be identified using the meniscal Flounce sign which is a fold in the free, non-anchored inner edge of the medial meniscus that can be noted during routine arthroscopy of the knee^9^. As per the literature, with all the other structures intact, there is no cue to identify the medial meniscus tear until checked with a probe under direct visualisation. An absence of the meniscus flounce sign is indicative of a hidden meniscal tear which needs to be addressed^9^.

Surgical modalities of repair differ from surgeon to surgeon and the nature of the tear. Broadly said, longitudinal and horizontal tears of the red-red zone and some tears of the red-white zone are considered repairable, while the others are best treated by meniscectomy. The repair can be done using inside out, outside in, or all inside techniques using various suture-based devices^10^. This said, an objective intra-operative sign indicating the adequacy of medial meniscal repair in such tears remains an enigma. To date, medial meniscus repair is deemed adequate only subjectively by different methods such as tugging at the meniscus with a probe^10^. This leaves a lot of room for error due to insufficient repair.

Primarily, the corresponding author performed surgeries on some cases and found a consistency in his finding of an S-shaped fold in the free, non-anchored, inner edge of the medial meniscus across all the cases. All the other surgeons involved in this study identified a similar S-shaped fold in the free, non-anchored, inner edge of the medial meniscus, formed simultaneously with the completion of an all-inside repair in hundreds of cases of isolated medial meniscus tears and subsequently validated the findings of the corresponding author. Consequently, we hypothesised that this S-shaped fold was an indication of adequate medial meniscus repair. It was labelled as the AMR (Adequacy of Medial meniscus Repair) sign, and this study was conducted to substantiate the hypothesis.

This novel, one of its kind studies, aims to address this nascent aspect of medial meniscus repair in knee arthroscopy by introducing an objective sign to denote the adequacy of repair of an isolated medial meniscus tear while using the all-inside technique. The other objective of our study was to assess whether this AMR sign can also be a reliable indicator of good functional outcomes and the risk of failure of repair in the longer run.

## Materials and Methods

Primarily, the corresponding author performed surgeries on some cases and found a consistent finding across most of the patients. This finding was then validated by four other surgeons at their hospitals. This multicentric, prospective study was hence conducted by five surgeons at five different hospitals and included 804 (n=804) consecutive cases with the clinical and radiological diagnoses of isolated medial meniscus tear (without concomitant injuries to the cruciates, lateral meniscus, or the collateral ligament), that warranted an arthroscopic repair. All procedures that were followed were in accordance with the Helsinki Declaration of 1975, as revised in 1983. The patients enrolled in this study were based on fixed inclusion criteria and were operated on between January 2014 and December 2017. Informed written consent of each patient was taken before enrolling them in the study.

Inclusion criteria for the study was patients with a history of locking and catching of the knee, tenderness over the medial joint line, positive Mcmurray’s sign clinically, patients whose MRI was suggestive of isolated medial meniscus tear, those with incidental finding of isolated medial meniscus tear on arthroscopy. Only patients with grade zero to grade two Osteoarthritis on radiographs (Kellgren Lawrence grading)^11^, and longitudinal or horizontal tears of the red-red zone or red-white zone, or bucket handle tears were included in the study.

Patients with a history of previous surgery in the affected knee, with a previous history of intra-articular fracture of the affected knee, with more than three degrees of varus deformity, with known collagen or storage disorders, who underwent an arthroscopic partial meniscectomy, with concomitant injuries to the chondral surface, lateral meniscus, cruciate ligaments or collateral ligaments, or with medial meniscus root tears were excluded from the study.

All the patients were assessed pre-operatively for clinical signs of medial meniscus injury by palpation over the medial joint line for tenderness, and McMurray’s test. This was followed-up by the radiological confirmation of the diagnosis. The Western Ontario Meniscal Evaluation Tool (WOMET) score and International Knee Documentation Committee (IKDC) score were calculated and maintained pre-operatively^12^. Type and location of tear were noted based on MRI findings. Power analysis revealed the power of the study to be 99%, keeping ^∝^ at 0.05.

**Fig 1:**
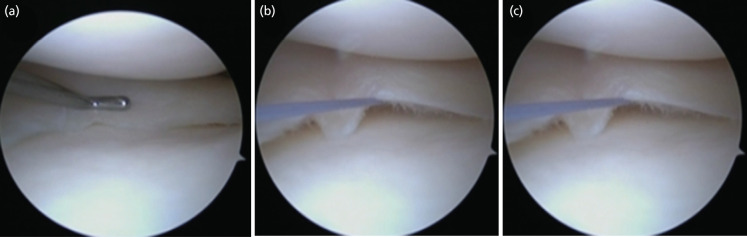
Case 71: (a) Medial meniscus tear with loss of meniscal flounce. (b) Formation of AMR sign after all inside repair. (c) The AMR sign.

In terms of the procedure that was carried out, patient preparation and diagnostic arthroscopic round were conducted on all the patients. Loss of medial meniscus integrity and the absence of meniscal flounce signs were confirmed. Location and type of tear were noted. Patients with horizontal or longitudinal tears in the red-red or red-white zone and bucket handle tears underwent repair of the medial meniscus with an arthroscopic all-inside repair technique. No augmentation (outside-in or inside-out) was performed prior to repair for any patient. Simultaneously, the AMR sign was noted in most of the patients (Fig. 1).

**Fig 2:**
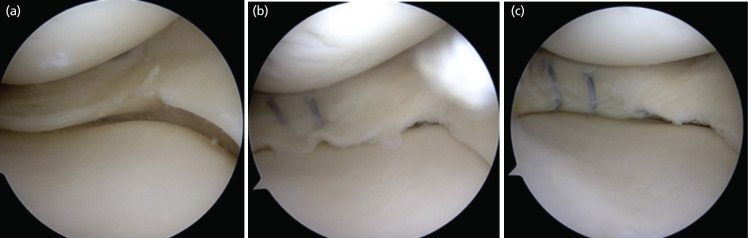
Case 128: (a) Before medial meniscus repair, (b) Formation of AMR sign after repair. (c) Loss of AMR sign on overtightening.

**Fig 3:**
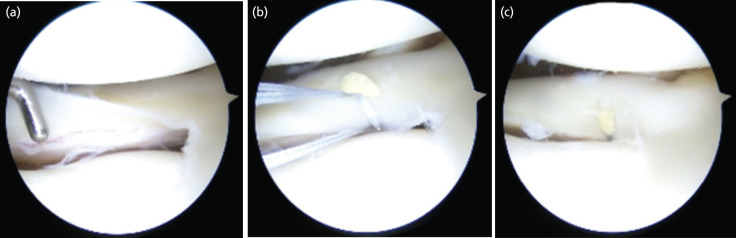
Case 467: (a) Horizontal medial meniscus tear in the red-white zone, (b) AMR sign not formed during repair. (c) No AMR sign on subjective completion of repair.

In cases where the AMR sign was formed and the surgeons subjectively felt the need for further tightening of the repair, the AMR sign was seen to disappear. This was noted in 40 (nl=40) out of the aforementioned 804 cases (Fig. 2). In 107 (nx=107) patients, the AMR sign was not noted on subjective completion of repair of the medial meniscus (Fig. 3), leaving us with 657 cases (nf=657) in which the AMR sign was noted. The formation of the sign was noted independent of the tear orientation (vertical or horizontal) or tear size among the sample size that fulfilled our inclusion criteria.

All the patients underwent supervised physiotherapy. Patients were advised non-weight bearing mobilisation for a period of three weeks, alongside quadriceps and Vastus-medius-obliquus strengthening and straight leg raises. After three weeks, graduated weight-bearing and gait-training were started. They were then followed-up closely and assessed at a mean duration of 42.34±4.537 months (Minimum: 30 months; Range: 30-55). The assessment at the terminal follow-up was done clinically by medial joint line tenderness and Mcmurray’s test and functionally using the WOMET and the IKDC. The WOMET being a disease-specific tool designed to evaluate health-related quality of life (HRQoL) in patients with meniscal pathology, we could clinically correlate the effect of our meniscal repairs with higher specificity^12^.

Additionally, the patients underwent an MRI scan at their terminal follow-up. MRI of the knee was evaluated by a single radiologist for all the cases, in a blinded manner. MRI confirmed the integrity of the repair by evaluating the meniscus shape, meniscal volume, meniscal signal, meniscal extrusion, and the intra-articular femoral cartilage status. If the patient had a positive McMurray’s test for medial meniscus, or medial joint line tenderness, or an MRI that was suggestive of either medial meniscus extrusion, increase in signal intensity at the previous site of tear^13^, loss of meniscus volume, or altered shape of the medial meniscus, the repair was considered to have failed.

All of the data was compiled and computed on the SPSS-24 software. Wilcoxon signed-rank test was done to analyse the changes in the pre-operative status and the status on terminal follow-up of the patient irrespective of whether the AMR sign was formed, lost after forming, or the AMR sign not formed. The Kruskal-Wallis test was applied to compare the functional outcome at terminal follow-up of the three groups (nf,nl, nx), and posthoc analysis was done for the intergroup comparison (nf vs nl and nf vs nx) of outcome scores at the respective terminal follow-up. Lastly, the Kruskal-Wallis test was used to analyse if there was any correlation between the failure of a repair and the intra-operative status of AMR sign.

## Results

The mean age of the study population was 28.58±5.878 years (Range: 17-43). Of the 804 patients included in the study, 482 (59.96%) were males and 322 (40.04%) were females. There was an almost equal predilection of the side affected with the left knee affected in 352 (43.82%) of patients and the right knee in 452 (56.18%) patients. Based on the AMR sign status intra-operatively, the patients were segregated into three groups, namely the AMR sign formed (nf=657), the AMR sign lost after formation (nl=40), and the AMR sign not formed (nx=107). The pre-operative evaluation of the patients was done using IKDC and WOMET scores. Tear morphology was recorded for each group. AMR sign formed group had 288 horizontal, 304 longitudinal and 65 bucket handle tears; AMR sign lost after formation group had 16 horizontal, 20 longitudinal and 4 bucket handle tears; AMR sign not formed group had 44 horizontal, 46 longitudinal and 10 bucket handle tears.

All patients were followed-up for a minimum of 2.5 years (30 months). The mean terminal follow-up of all the patients was recorded as 42.34±4.537 months (Range: 30-55) after the procedure. At the terminal follow-up, all the patients were re-evaluated functionally using the WOMET and IKDC score. Wilcoxon signed-rank test for non-parametric longitudinal evaluation of the change between the preoperative and post-operative scores was done, and it was found that the patients in all the three groups showed a statistically significant (p<0.05) improvement at their terminal follow-up from their pre-operative IKDC and WOMET scores. The application of the Kruskal-Wallis test was done to the three groups in order to compare the overall mean of all the parameters based on the H value and keep a degree of freedom (df) as two. Comparing mean overall IKDC out of 87 (Pre-op vs Post-op: 35.99±4.582 vs 79.76±3.683) and mean overall WOMET out of 100 (Pre-op vs post-op: 46.35±5.543 vs 10.27±4.712), it was found that the difference between the overall pre-operative and terminal scores was significantly better (p<0.05) at the follow-up, suggesting good outcome in all patients, irrespective of the surgery performed (Table I).

**Table I:** Longitudinal intra-group comparison between pre-operative score and terminal follow-up score of mean IKDC* and mean WOMET# of the three groups using Wilcoxon Signed Rank Test (p value <0.05 = significant)

Group	Mean IKDC (Standard Deviation)				Mean WOMET (Terminal Deviation)			
	Pre-op	Terminal follow-up	Z value	*p* value	Pre-op	Terminal follow-up	Z value	*p* value
AMR sign formed	36.032	80.125	22.217	<0.01	46.41	9.82	22.212	<0.01
(nf=657)	(4.572)	(3.742)			(5.631)	(4.791)		
AMR sign lost	35.475	77.925	5.519	<0.01	46.525	12.4	5.513	<0.01
(nl=40)	(4.852)	(2.895)			(5.652)	(3.774)		
AMR sign not formed	35.888	78.196	8.985	<0.01	45.9	12.22	8.974	<0.01
(nx=107)	(4.575)	(2.909)			(4.949)	(3.753)		

*IKDC: International Knee Documentation Committee #WOMET: Western Ontario Meniscal Evaluation Tool

To ascertain the clinical implications of finding the AMR sign intra-operatively during meniscal repair, the value of the functional outcome scores of the three groups at terminal follow-up were subjected to a Kruskal-Wallis analysis, keeping the degree of freedom as two and the cut-off for statistical significance as a p-value <0.05. The H-value for IKDC and WOMET score at the terminal follow-up were computed to be 33.677 and 30.669, respectively (p-value <0.05). This meant that the AMR sign formed group had a significantly better functional outcome compared to its counterparts (Table II).

**Table II:** Comparison of mean IKDC* and WOMET# scores at the terminal follow-up of the three groups using Kruskal-Wallis Test (p value <0.05 = significant; degree of freedom: 2)

Group	Mean IKDC* (Standard Deviation)				Mean WOMET# (Terminal Deviation)			
	Pre-operative	Terminal follow-up	H value	p value	Pre-op	Terminal follow-up	H value	p value
AMR sign formed	36.032	80.125	33.677	0.00	46.41	9.82	30.669	0.00
(nf=657)	(4.572)	(3.742)			(5.631)	(4.791)		
AMR sign lost	35.475	77.925			46.525	12.4		
(nl=40)	(4.852)	(2.895)			(5.652)	(3.774)		
AMR sign not formed	35.888	78.196			45.9	12.22		
(nx=107)	(4.575)	(2.909)			(4.949)	(3.753)		

*IKDC: International Knee Documentation Committee #WOMET: Western Ontario Meniscal Evaluation Tool

Clinical evaluation was done by checking for medial joint line tenderness and pain on Mcmurray’s test for medial meniscus. Additionally, Radiological evaluation was done using a fresh MRI of the operated knee, and assessed for medial meniscus extrusion, increase in signal intensity at the previous site of the tear, loss of meniscus volume or altered shape by the radiologist. The presence of even one of the two clinical or four radiological criteria was considered as a failure of repair.

Finally, the association between AMR signs and the risk of failure of repair was assessed. The total number of failures was 52 (6.47%). The distribution based on groups and aetiology was as shown in (Table III). Upon application of the Kruskal Wallis test, keeping the df as two, it was noted that the number of repair failures was higher in groups where the AMR sign was not formed or lost after formation, as compared to when the AMR sign was formed. This association was statistically significant (H-value: 7.938 and p-value: 0.019 (<0.05)). Additionally, the Mann-Whitney U test confirmed the accuracy of MJL tenderness, McMurray’s test and the MRI criteria as efficient tools for identifying failure of repair even independently.

**Table III:** Group wise distribution of number of failures and the association using Kruskal-Wallis test. (p value <0.05 = significant; df=2)

Group	Failure of repair	Etiology		Mean rank	H value	p value
		Atraumatic	Traumatic			
AMR sign formed (nf=657)	35 (5.33%)	10 (28.57%)	25 (71.43%)	407.08	7.938	0.019
AMR sign lost (nl=40)	4 (10%)	2 (50%)	2 (50%)	388.30		
AMR sign not formed (nx=107)	13 (12.155)	4 (30.77%)	9 (69.23%)	379.66		
Overall	52 (6.48%)	16 (30.77%)	36 (69.23%)			

## Discussion

There has been an ongoing shift in the trend of management of medial meniscus tears, with a growing understanding of the important roles it plays in joint biomechanics. The modern orthopaedic literature has emphasised its vitality in maintaining joint integrity and cushioning the effects of axial loading activities. With the earlier school of thought more inclined towards excision of the damaged medial meniscus, there was a higher susceptibility of these knees to developing early osteoarthritis. Hence, orthopaedic surgeons have now moved from resection to preservation, repair, and even reconstruction of the meniscus with more importance given to preservation and repair of the native medial meniscus^1^.

**Fig 4:**
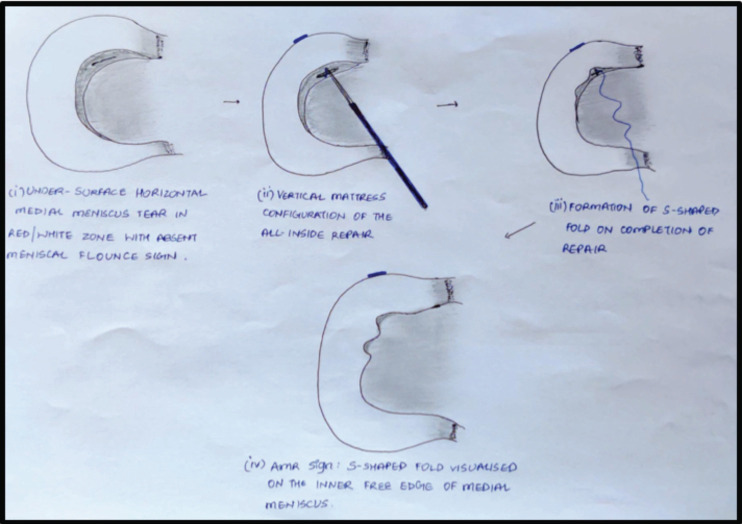
Diagrammatic illustration of the AMR sign showing (clockwise) (i) Undersurface horizontal tear of the medial meniscus in red-white zone with absent meniscal flounce sign (ii) Vertical mattress configuration of all inside arthroscopic repair of the tear (iii) Formation of the S-shaped curve in the inner, free, non-anchored edge of the medial meniscus upon completion of the repair (iv) Persistence of the AMR sign as visualised arthroscopically.

This increase in the number of arthroscopic repair surgeries opened the gates to accompanying sequelae such as inadequate repair or tensioning of the meniscus. With the lack of an objective, definitive sign to suggest an adequate repair, the surgeons rely on subjective assessment of the strength of fixation by probing the meniscus after a repair which has a high possibility of inter-observer error and bias. Therefore, in this first of its kind study, we aimed to introduce a novel arthroscopic sign that is a definitive indication of an adequately repaired medial meniscus, namely, the AMR sign (Fig. 4). The formation of this sign was studied in detail to evaluate not only its intra-operative significance but to also establish a clinical-radiological correlation between attaining this sign and good long-term outcomes.

The demographic distribution of our study population in terms of mean age, gender, and side affected were congruent with the general findings in the orthopaedic literature^1,2,13^. For the functional evaluation of the patients pre-operatively and on terminal follow-up, we used the IKDC score due to its reproducibility and universal acceptance, and the WOMET score due to its specificity for medial meniscus pathologies^14^.

The mean overall pre-operative IKDC score of our study population was 35.99±4.582 which was similar to the findings of Nezhad and Navali in 201715, and Abdallah et al in 202016. The patients were divided into three groups. The first group had those patients in whom the AMR sign was formed and was maintained. The second group was one in which the AMR sign was formed and then lost, presumably due to overtightening. The third group was the one in which the AMR sign was not formed.

Staying consistent with the results of Hopkins *et al*^17^, our patients too showed a significant improvement (p<0.01) in their functional outcome scores, irrespective of the type of injury and aforementioned group, as was shown by the Wilcoxon signed-rank test.

Once the AMR sign has formed, the hoop strength necessary for the transmission of weight from the femur to the tibia is adequate. If further tightening is attempted, it acts via the meniscocapsular attachment, increasing the hoop strength and stretching the meniscus out. This presumably leads to the loss of the AMR sign. 72.5% (29/40) of the patients in whom the AMR sign was lost following formation had significant post-operative pain, as well as continued intermittent pain owing to capsular tightening. However, as the number of cases in our study with capsular tightness was considerably smaller as compared to the entire sample size, it is difficult to extrapolate this finding and needs further research. Hence, we presume this sign is a crucial indicator to the surgeon with regards to the endpoint of the fixation such that it is neither inadequate nor overtightened. The AMR sign formed group had statistically insignificant, albeit better IKDC and WOMET scores compared to the AMR sign lost group, while both of these groups had better functional scores than the AMR sign not formed group which was also statistically significant.

A total of 52 of our patients (6.5%) had repair failure, with the majority of them being secondary to another episode of trauma. This was similar to the lower limit of failure rates reported all around the world and in the meta-analysis of Ow *et al* (2021)^18^. These failures were diagnosed either clinically or radiologically using an MRI scan, based on the criteria set by our radiologist and derived from the literature^13^. Being a multicentric study which was conducted by five surgeons in five different centres, the reproducibility of the AMR signs can be vouched for. Although a second look arthroscopy would’ve been the gold standard for the diagnosis of repair failure, none of the patients agreed on the same and this is hence one of the drawbacks of the study which can be worked on.

We encourage more research on the application of the AMR sign in varied ages, geographical distribution, types of injuries, and techniques of repair, with higher sample size, and a longer mean follow-up period. Nevertheless, based on the results of the study population that we procured; the AMR sign is potentially a pathbreaking tool in the arsenal of an orthopaedic surgeon striving for the adequate repair of an isolated medial meniscus tear.

Meniscus surgery has come a long way from the old slogan, ‘if it is torn, take it out!’ to the currently accepted ‘Save the meniscus!’, which guides the evolving modern treatment methods for meniscal tears^19^. With increasing meniscus repair surgeries, identifying hidden tears and sufficient fixation of the repair is the need of time. The AMR sign is a consistent landmark sign of the medial meniscus hoop strength and intactness of its collagen architecture post repair.

## Conclusion

Medial meniscus repair adequacy is usually checked by orthopaedic surgeons by probing and tugging at the meniscus post-repair to gauge its tension subjectively, leaving it susceptible to inadequate repair, especially by the budding surgeons. AMR sign is a nascent arthroscopic sign that is formed after an adequate repair of the medial meniscus. It is an S-shaped fold in the inner, non-anchored, free edge of the medial meniscus. Not only is it a landmark sign for objectively confirming an adequate medial meniscus repair, but its consistency ensures excellent long term functional, radiological and clinical outcomes in the patient. The formation of the AMR sign is thereby a reliable guide to orthopaedic surgeons all across the globe in dealing with the arthroscopic repair of isolated medial meniscus tears.
